# An Ensemble Analysis of Electromyographic Activity during Whole Body Pointing with the Use of Support Vector Machines

**DOI:** 10.1371/journal.pone.0020732

**Published:** 2011-07-26

**Authors:** Arvind Tolambiya, Elizabeth Thomas, Enrico Chiovetto, Bastien Berret, Thierry Pozzo

**Affiliations:** 1 Université de Bourgogne, Campus Universitaire, BP 27877, F-21078 Dijon, France, INSERM, U887, Motricité-Plasticité, Dijon, F-21078, France; 2 Section for Computational Sensomotorics, Department of Cognitive Neurology, Hertie Institute for Clinical Brain Research, Centre for Integrative Neuroscience, University Clinic Tübingen, Tübingen, Germany; 3 Italian Institute of Technology, Genoa, Italy; 4 IUF, Université de Bourgogne, Campus Universitaire, BP 27877, F-21078 Dijon, France; University of Sheffield, United Kingdom

## Abstract

We explored the use of support vector machines (SVM) in order to analyze the ensemble activities of 24 postural and focal muscles recorded during a whole body pointing task. Because of the large number of variables involved in motor control studies, such multivariate methods have much to offer over the standard univariate techniques that are currently employed in the field to detect modifications. The SVM was used to uncover the principle differences underlying several variations of the task. Five variants of the task were used. An unconstrained reaching, two constrained at the focal level and two at the postural level. Using the electromyographic (EMG) data, the SVM proved capable of distinguishing all the unconstrained from the constrained conditions with a success of approximately 80% or above. In all cases, including those with focal constraints, the collective postural muscle EMGs were as good as or better than those from focal muscles for discriminating between conditions. This was unexpected especially in the case with focal constraints. In trying to rank the importance of particular features of the postural EMGs we found the maximum amplitude rather than the moment at which it occurred to be more discriminative. A classification using the muscles one at a time permitted us to identify some of the postural muscles that are significantly altered between conditions. In this case, the use of a multivariate method also permitted the use of the entire muscle EMG waveform rather than the difficult process of defining and extracting any particular variable. The best accuracy was obtained from muscles of the leg rather than from the trunk. By identifying the features that are important in discrimination, the use of the SVM permitted us to identify some of the features that are adapted when constraints are placed on a complex motor task.

## Introduction

Studies on motor control generate a huge amount of data involving a large number of variables. For example the whole body pointing task being explored here involves electromyographic (EMG) recordings from 24 different muscles each of which can be characterized by at least 3 different variables (e.g. maximum amplitude, onset time, time to peak). Electromyographic data that yields much insight on motor control is also known to contain a high amount of inter and intra subject variability [1], [2]. In this study we investigate how support vector machines can be utilized in the analysis of electromyographic data underlying a whole body pointing task. This is a complex, multijoint task involving several segments of the body.

Despite the importance of such types of movement in our daily lives, most previous studies of human motor control either focus on the equilibrium mechanisms primarily involving the postural component (for review, see [3]) or conversely on reaching movements of the focal module while restricting motion of the lower body part (for review, see [4]. The study of movements involving both components however would help us to understand the role and interactions of the postural elements with the focal modules. The former is thought traditionally to be controlled by lower brain structures with commands primarily conveyed via the ventromedial pathway while the latter is thought to require intervention by the motor cortex via the lateral pathway [5], [6].

**Figure 1:**
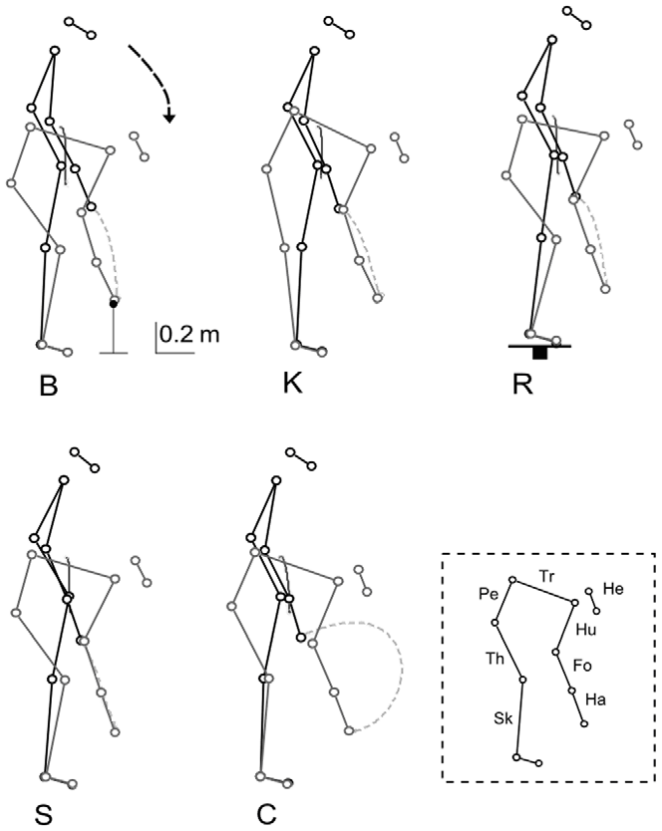
Stick diagrams of the task performed under basic condition (B), equilibrium constraints (K, R) and spatial constraints (S, C). B: Basic condition. K: Knee-extended condition. R: Reduced base of support condition. S: Imposed straight finger trajectory condition. C: Imposed semicircular trajectory condition. The dark gray and light gray dotted traces depict the CoM and the finger trajectories in the sagittal plane, respectively. The inset box defines the body parts in the stick diagram (Sk, shank; Th, thigh; Pe, pelvis; Tr, trunk; He, head; Hu, humerus; Fo, forearm; Ha, hand).

One way to extend our understanding of the whole body pointing movement is to analyze the difference between several variants of it. Movement is thought to take place through the use of a core program which is then adjusted to meet the necessities as they arise [7]. We therefore examined several variants of a whole body pointing task. It can be accomplished in several different ways based on the limits imposed by the environment. Some of them might require alterations in balance control such as when we have to reach to an object from a reduced base of support. A higher obstacle or limited upper reaching space on the other hand could call into play more adjustments at the focal level. We therefore studied one unconstrained whole body pointing (B), two types of pointing with focal constraints and two with postural constraints. The postural constraints were a reduced base of support (R) and an extended knee condition (K). For the focal constraints we imposed a straight finger trajectory (S) or a semicircular finger trajectory (C) (figure 1). These tasks therefore represent several possibilities for the hand and centre of mass trajectories in sagittal space.

Although they have much to offer in terms of understanding human movement, such studies remain difficult in part because of the challenges involved in the analysis of the voluminous dataset that is produced. One way to approach the task is to create physiologically meaningful ensembles of the muscles and to probe if they have undergone significant alterations between movement conditions. Such methods also bring with them the potential benefits of a multivariate technique. We approached the problem by using a classification paradigm in order to probe the differences between constrained and unconstrained pointing movements. The SVM was used to carry out the classification. It belongs to the class of kernel methods which in turn belongs to the larger class of machine learning techniques. The latter term obtains its name from the use of part of the data as a training set in order to find the surface that best separates two classes of data. A test set is then used to verify if the constructed surface is also able to correctly classify data that had not been used for training. The inability to correctly distinguish the test data sets indicates the lack of sufficient differences to distinguish the data sets being classified. A larger separation between the data sets would lead to a greater ease and success of classification.

Two common classes of machine learning techniques are neural networks and the kernel methods. The SVM was used in this investigation because several studies have now demonstrated that the kernel methods are more efficient than neural networks for classifying or in other words differentiating two classes of physiological data. The kernel methods have also been found to have higher classification accuracies than linear classification methods like linear discriminant analysis [8], [9], [10]. Any unsupervised clustering methods such as classical cluster analysis methods or unsupervised neural networks [11], [12] would be unsuitable as we sought in this study to identify the muscle groups before the start of the analysis.

This study is an extension of three previous investigations on whole body pointing [13]–[15]. In this manner Berret et al [13] were able to represent with two principal components, all the variability from the recordings of 8 kinematic angles during the pointing movement. Three principal components were found sufficient to capture the information from 24 muscles recorded during the same movements [14], [15]. While focusing on dimensionality reduction in such a hyper redundant musculoskeletal system, these studies did not reveal the muscles that could be significantly modified by the task demands. This is because such techniques were not developed for detecting first order differences between datasets but rather for extracting common features. They are based on the covariation between the datasets while most of the machine learning techniques are based on Euclidean distances. The covariation between muscular activities however can stay unchanged even as important alterations take place in individual or collective EMG timings and amplitudes. Alterations observed in this compressed data space can also be very difficult to interpret due to factors such as the participation of one muscle in more than one group. The techniques mentioned above therefore present several limitations when it comes to the question of identifying differences. Classification techniques such as neural nets and kernel methods (SVMs belong to this group of methods) permit a larger degree of control on the ensembles created and hence an easier interpretation of their alterations. The presented classification techniques therefore create a complement to the understanding of motor control that can be obtained by methods such as PCAs, non negative matrix factorization or independent components analysis [13]–[18].

The first task undertaken in this study was to probe if the SVM was able to discriminate using EMG data from the 24 muscles, the type of movement that had been undertaken. Following some success with this attempt, we then undertook the same types of classifications using either postural or focal muscles. We expected that the former would be more predictive when the constraints were postural. Since no marked changes had been observed during the kinematic studies, we hypothesised that the postural muscles would be quite poor at classifying the conditions with focal constraints. In all cases however, classification attempts using data from the postural muscles were as or more successful than those obtained using focal muscles. This indicated that between conditions, greater discriminating differences could be found at the postural rather than at the focal level. We then proceeded to analyse the importance of particular EMG characteristics for the classification. The importance of postural muscle EMG amplitude as opposed to its temporal characteristics was probed by attempting a classification with input vectors only containing these extracted features. As in the case of gait [17] would the timing of the EMG bursts be a relatively invariant parameter between the different types of pointing? In this case, we would be unable to classify a movement with this parameter. The last step in the investigation involved a classification attempt using one postural muscle at a time. Once again, this would indicate to us which type of muscle – axial, proximal or distal, is tuned and adapted to the movement at hand.

## Methods

The study was carried out by analyzing electromyographic data obtained from 24 muscles while the subject carried out several different types of reaching movements. Comparisons between groups were carried out by attempting a classification task with the SVM. Detailed descriptions of the experimental conditions for the study have been provided in three previous reports. Information concerning the gathering of the kinematic data and its analysis can be found in Berret et al [13]. Reports by Chiovetto et al [14] and Fautrelle et al [15] provide more information on the procedures concerning the collection of EMG data. As this is a follow up study, we will first provide a general description of the experimental conditions. As kinematic data in this study was only used to interpret the EMGs, only a brief mention will be made of this aspect. This will then be followed by a more detailed description of the EMG data gathering and SVM analysis.

### General

Data from ten healthy male subjects (ages 29±4 years) were used in this study. They had participated voluntarily in the experiment. All subjects were in good health and had no previous history of neuromuscular disease. The experiment conformed to the declaration of Helsinki and informed consent was obtained from all the participants according to the protocol of the local ethical committee.

Participants were required to point with both their index fingers at the extremities of a wooden dowel located in front of them. All movements were self paced. The dowel was positioned horizontally with respect to the ground, parallel to the subjects' coronal plane and with its centre intersecting the subjects' sagittal plane. For each participant, the extremities of the dowel had a vertical distance from the ground equal to 15% of their body height. The target distances (measured starting from the distal end of the participants' great toe) corresponded to 5% of participants' height. The subjects performed a pointing task towards this target under unconstrained (B), knee extended (K), reduced base of support (R), imposed straight finger trajectory (S) and finally imposed semicircular finger trajectory (C) conditions (figure 1). For the B condition, participants started from an upright standing position with their hands initially located at the external side of the thighs and then executed hand-pointing movements in a semipronated position. The whole movement was assumed to be symmetrical [13] and was performed in the sagittal plane with each side of the body moving together. Target accuracy was not the primary constraint during the experiments and no instruction was given to the participants regarding the strategy to follow in accomplishing the task.

The B movements were the only ones performed without any constraints. Postural constraints were imposed for the K and R conditions. In the K conditions, subjects were instructed to point to the target without flexing the knees. In the R conditions, reaching movements were made from a reduced base of support – a square wooden board 40×40 cm^2^. Subjects were able to perform both types of tasks without losing their balance.

Focal constraints were applied for the S and C conditions. In the S conditions, participants were asked to point to the targets by using a straight finger trajectory. Participants initially performed three nonrecorded trials by following a straight wire connecting the initial finger position to the target. After this short period, they were asked to perform the task without wire. In the C conditions, participants were requested to reach the targets with large finger path curvatures (semicircular finger trajectory). The imposed path was concave in the sagittal plane. Once again, the participants performed three nonrecorded trials by tracking a curved wire connecting the initial finger position and the targets. They were then asked to perform the task without wire.

During trial executions, kinematic and EMG data were simultaneously monitored. Body kinematics was recorded by means of a Vicon (Oxford, UK) motion capture system. Finger kinematics was used in order to define basic parameters in the finger pointing. These parameters have been well defined in a previous study of arm-pointing [19]. Finger movement onset time *t_o_*, was defined as the instant at which the linear tangential velocity of the index fingertip exceeded 5% of its peak. The end of the movement *t_f_* was the point at which the same velocity dropped below the 5% threshold.

### Collection of electromyographic data

**Figure 2:**
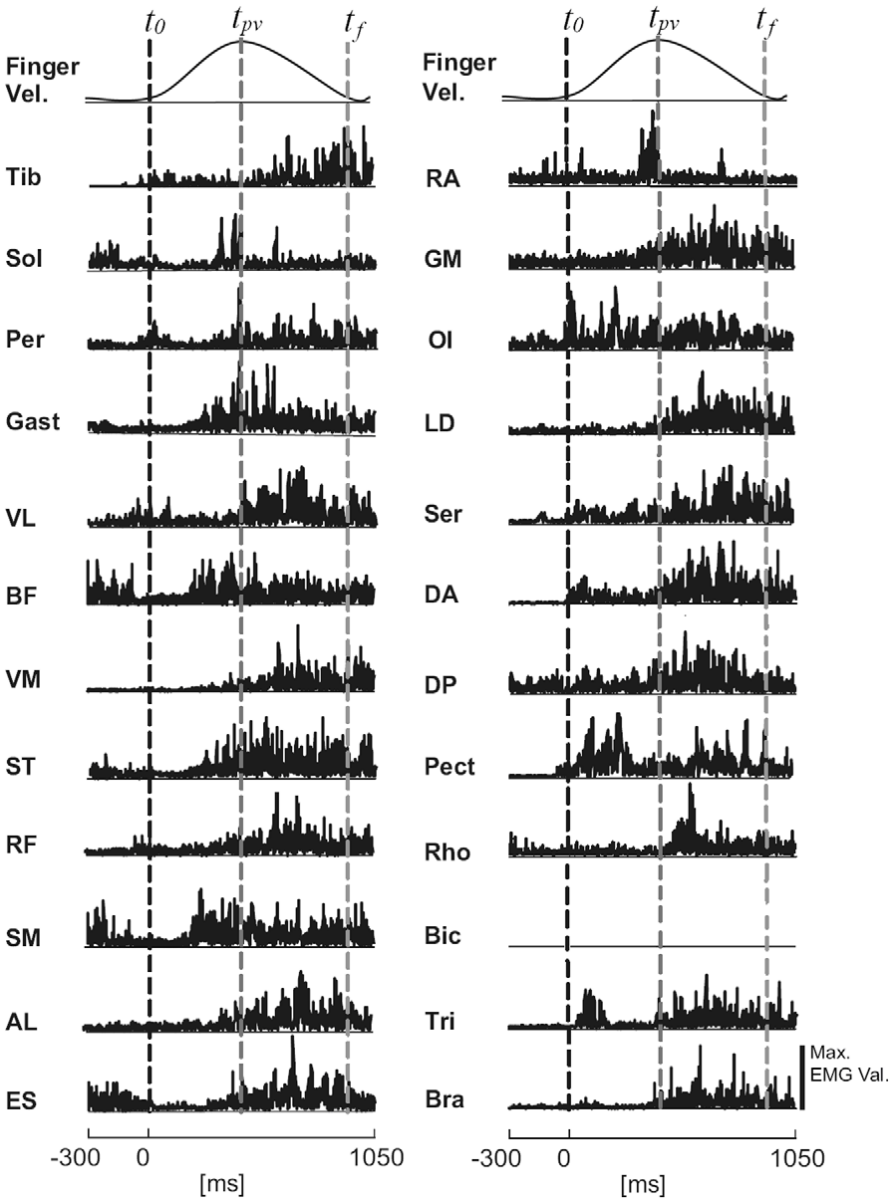
EMG recordings. Traces of the 24 muscles recorded from an individual during a whole body pointing task. Muscle abbreviations are explained in the Methods section. Recordings from the Bic are missing due to difficulties with the electrode for this individual. The EMG signals presented were recorded at a sampling frequency of 1000 Hz followed by rectification. The first trace in each column represents finger velocity. Finger movement onset is indicated by *t_o_*, the instant of its maximum velocity by *t_pv_* and the instant of finger movement termination by *t_f_*.

The following 24 muscles were recorded on the right side of each of the 10 subjects: tibialis anterior (Tib) ; soleus (Sol) ; peroneus longus (Per) ; gastrocnemius (Gast) ; vastus lateralis (VL) ; vastus medialis (VM) ; rectus femoris (RF) ; semitendinosus (ST) ; semimembranosus (SM) ; biceps femoris (long head) (BF) ; adductor longus (AL) ; gluteus maximus (GM) ; rectus abdominis, superior portion (RA) ; internal oblique (OI) ; erector spinae, recorded at L2 (ES) ; (these fifteen first muscles will be referred to as “postural” muscles in our task); serratus anterior (Ser); pectoralis, superior portion (Pect); latissimus dorsi (LD); rhomboid (Rho); deltoideus anterior and posterior portions (DA and DP, respectively) ; biceps brachii (Bic); brachioradialis (Bra); and triceps brachii (Tri) (these nine last muscles will be referred to as “focal” muscles). For all these muscles, electrodes were placed to minimize cross talk from adjacent muscles contractions following Ivanenko et al. [17] guidelines. The interval between a pair of electrodes for one recorded muscle was set to two centimeters. In order to check the goodness of electrodes location, the subjects were instructed how to selectively activate each muscle [20] and the experimenter could verify the signal response on a computer screen. During preparation, subjects' skin was shaved and cleaned with alcohol to ensure low resistance. Then the surface EMG activities were recorded at a sampling frequency of 1000 Hz (figure 2) (ZERO WIRE EMG system, AURION S.r.l., Milano, Italia). Each electrode was equipped with a little unit for signal processing and 6 tele-transmissions. The EMG signals were rectified and then smoothed using a Butterworth filter with a cut-off frequency at 5 Hz [1]. Details concerning the amplitude normalization of the EMGs can be found in the sub-section ‘Construction of the input vectors’ of the Methods section. The movement duration was normalized to 200 points (figure 2).

### Classification with the SVM

A multivariate comparison of the EMG data collected from 24 muscles during the various reaching tasks was carried out using the SVM. Success by the SVM in a categorization task indicated the presence of sufficient differences between two groups. In this section we will provide first of all, a succinct description of SVMs. This will be followed by a description of the manner in which the input vectors were created for the SVM. We will then describe the method used for creating the training and testing samples.

#### Support Vector Machines

**Figure 3:**
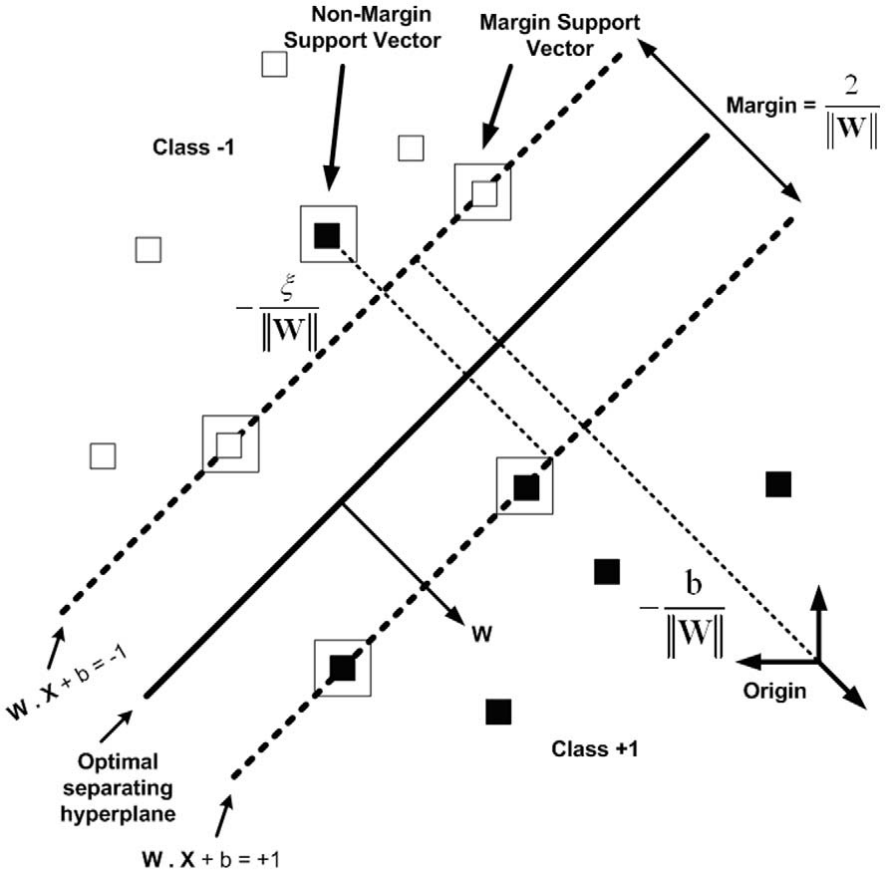
Optimal separating hyper-plane in SVMs for a linearly non-separable case. Black and white squares refer to the classes ‘+1’ and ‘−1’, respectively. Support vectors are indicated by an extra square.

Support vector machines (SVMs) are powerful methods for solving classification problems on large datasets. SVMs originated from statistical learning theory and are based on the principle of structure risk minimization (SRM). SVM was first developed by Vapnik and his co-workers in the early 1990s. In a binary classification task, SVM aims to find an optimal separating hyperplane (OSH). Another aspect of SVMs is the transformation of data into higher dimensional space for the construction of the OSH. SVMs perform this nonlinear mapping into a higher dimension feature space by means of a kernel function and then construct a linear OSH between the two classes in the feature space (figure 3). Thus, although it uses linear learning methods due to its nonlinear kernel function, it is in effect a nonlinear classifier. A complete formulation of Support Vector Machines can be found in a number of publications [21]–[25]. Here, the brief theory of SVMs for nonlinear classification will be presented.

Let us consider a supervised binary classification problem. Let us assume that the training set consists of *N* vectors from a *d*-dimensional feature space 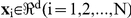. A target 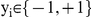 is associated with each vector 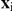. Searching an OSH in the original input space is too restrictive in most practical cases. In SVM, nonlinear classification problems are solved by mapping the original data 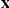 into a higher dimension feature space 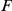 by 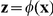 via a nonlinear mapping 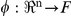, in which the mapped data are linearly separable. Considering the case when the data are linearly nonseparable in 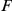, there exists a vector 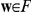 and a scalar 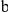 that define the separating hyperplane as: 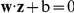 such that
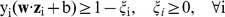
(1)where the 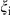
's are the *slack variables* introduced to account for the nonseparability of data.
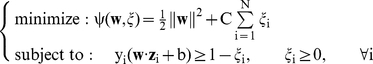
(2)


The constant C represents a regularization parameter that controls the penalty assigned to errors. The larger the C value, the higher the penalty associated with misclassified samples. This optimization problem can be translated into a dual problem using a Lagrangian formulation as follows:
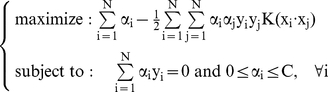
(3)where 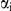 are the nonnegative Lagrangian multipliers. The data points 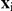 corresponding to 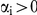 are the support vectors. It is worth noting that, in the nonseparable case, two kinds of support vectors coexist: (a) margin support vectors that lie on the hyperplane margin and (b) nonmargin support vectors that fall on the “wrong” side of this margin (figure 3). The kernel function 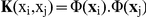 satisfies the Mercer's condition [25] and can be computed without having explicit knowledge of 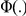. For any test vector 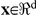, the output is then given by:
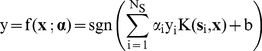
(4)


Where, the 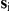 are the 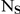 support vectors. To build an SVM classifier, the user needs to tune C and choose a kernel function and its parameters. The performance of the SVM is very closely tied to the choice of the optimal kernel functions. There has been a lot of research over the last few years on algorithms to help choose the exact type of kernel for a given problem with a certain set of features. Most of these methods depend on simple heuristics that are based on the knowledge of the input data. There has not been any standardized method to obtain the best kernel. Hence, the choice of the optimal kernel has been reduced to a trial and error procedure in most scenarios. There exist many popular kernel functions that have been widely used for classification e.g., linear, Gaussian radial basis function, polynomial and Wigner kernel. In this study, we experimented with different kernels. We found the Wigner kernel to be best suited for our problem. The Wigner kernel is defined as:
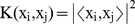
(5)


The notation 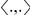 indicates an inner product.

The SVM analysis was carried out with a MATLAB program that had been written and utilized in two previous studies [26], [27].

#### Construction of the input vectors

The construction of the input vectors for the SVM depended on the comparison at hand. The EMG data from each muscle constituted a vector of 200 elements (movement duration was normalized to 200 points. A comparison using all 24 muscles, was therefore done using an input vector of size (24×200) where the input vectors of the muscles were linked together from end to end. This manner of constructing the input vectors for a classification of EMG data has already been described in Nair et al [8]. In other words the entire rectified, filtered and normalized EMG waveform (as described in the section ‘Collection of electromyographic data’) of each muscle was utilized without any particular feature extraction. In this manner we hoped to put in place a method whereby we would be more directly able to identify the features that were essential to the classification. There are several techniques that have been developed for this process of feature extraction [28]. An exploration of these methods was considered beyond the scope of the current study but our future investigations will involve testing them.

Subsets of these input vectors were used when the questions addressed involved only specific types of muscles or specific instances during the movement. This therefore led to the creation of a *focal muscle vector* or a *postural muscle vector* based on the question at hand. For the results displayed in Table 1, specific features were extracted from each muscle. In this case, the contribution from each muscle consisted only of 1 element, either the maximum amplitude or the time at which this occurred. This information from all the postural muscles was then put together to create either the *maximum amplitude vector* consisting of the maximum EMG amplitudes from 15 postural muscles or the *maximum time point vector*, with the timing of these maxima.

**Table 1:** Classification accuracies using the *maximum amplitude vector* and the *maximum time point vector*.

	Mean values for the percent of correct answers above chance (%)		
	*maximum amplitude vector*	*maximum time point vector*	p
B vs K	23	17.54	p>0.05
B vs R	16.17	6.78	p<0.05
B vs S	27.65	−8.38	*p<0.01
B vs C	18.93	3.64	*p<0.01

Mean values of correct responses obtained for classification between constrained and unconstrained conditions. Values are reported as percentage above chance levels. The asterisk marks cases for which the categorization success using the *maximum amplitude vector* was significantly higher than with the *maximum time point vector*. Statitical tests were carried out using a Friedmann test followed by the Wilcoxon tests with a Bonferroni correction for multiple comparisons.

Input vectors for the SVM were normalized. The normalization was carried out over each muscle and each individual so that the information from each muscle carried equal importance. For each muscle however, information concerning the amplitude differences for each movement condition was available as the normalization was carried out by linking together the EMGs for the two types of movements being compared.

#### Data sampling

For each type of movement, each subject was represented by 6 trials. The classification tasks were carried out using 5-fold cross validation. For each study we divided each group into 5 folds (The input vectors from two subjects in each fold). Four folds were used for training and the last fold kept for testing. *At no point in these studies was the data from individuals that were used for training, used in testing*. This process was repeated 5 times, leaving one different fold for evaluation each time. The percentage of correct classification was verified for each subject when they were in the test case. Accuracy rates were reported as the mean over all 10 subjects.

#### Statistical significance

Two types of tests for statistical significance were done. In cases where a parametric test was appropriate they were either a one way repeated measures ANOVA followed by a Tukey HSD post-hoc test. In cases where parametric tests were inappropriate we applied a Friedmann test followed by the Wilcoxon tests with a Bonferroni correction for multiple comparisons.

#### The κ coefficient

The κ coefficient is a measure of of the agreement between two judges concerning the label to be assigned to the data. It quantifies how well the classification had been performed by comparing the results obtained from the SVM with the correct answers [29]. The calculation is based on the difference between how much agreement is actually present (“observed” agreement) compared to how much agreement would be expected to be present by chance alone (“expected” agreement). This difference is standardized to lie on a −1 to 1 scale, where 1 indicates perfect agreement, 0 is exactly what would be expected by chance, and negative values indicate agreement less than chance, i.e. potential systematic disagreement with correct answers. The following values of κ have been taken to indicate various levels of agreement between the automatic classifier and the correct answer. Values of κ<0 no agreement, 0<κ<0.2 slight agreement, 0.21<κ<0.4 fair agreement, 0.41<κ<0.6 moderate agreement and 0.61<κ<0.8 substantial agreement, 0.81<κ<1.0 Almost perfect agreement. The value of κ is defined as
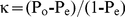
(6)


Where P_o_ is the observed level of agreement between the two classifiers and P_e_ is the agreement that could be expected from two individuals flipping a coin to assign a class label.

## Results

Several binary classifications with the SVM were undertaken in order to gain insight into alterations in the 24 muscles that produced the different pointing movements. We first show that the SVM is capable of discriminating the B movements from the movements with constraints. We then compare the capacities of the *focal muscle vector* and *postural muscle vector* for classifying the movements. A higher capacity for classification using a particular vector is due to greater discriminating differences between the conditions. Another way of phrasing this would be to say that a poor classification indicates a greater overlap between the datasets being classified. In the following section the same classification tasks were carried out using extracted features from the muscle EMGs. These were either the *maximum amplitude vector*s or the *maximum time point vector*. After having found that classification with the *postural muscle vectors* was in all cases equal to or better than that obtained with the *focal muscle vector*, we finally undertook a muscle by muscle investigation of the capacities of the postural muscles for discriminating certain experimental conditions.

### The SVM is capable of discriminating the EMGs from different movements

**Figure 4:**
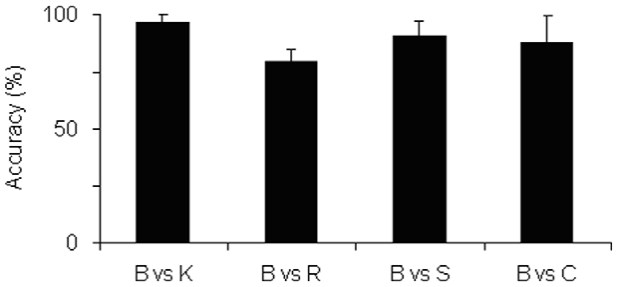
All muscle binary classifications. Binary classification of the unconstrained B condition against the constrained K, R, S and C movements using the EMG data from all 24 muscles.

Our first task was to ensure that the SVM was capable of discriminating the EMGs that had produced the different types of whole body pointing. Four different binary classifications each time to distinguish the B movement EMGs from those of the constrained movements were undertaken (figure 4). The data from all 24 muscles was used for these tasks. Our results show that in all cases, the SVM was able to distinguish the constrained from unconstrained movements with a mean success rate close to or higher than 80%. The lowest classification success was obtained for discriminating the R condition at a mean accuracy of 79.4%. The remaining K, S and C conditions could be separated from the B condition with mean accuracy rates of 96.7%, 90.6% and 87.6% respectively.

The values of the κ coefficient from these classifications also show that the SVM is capable of exploiting the differences in the EMGs produced from the different types of whole body pointing. They were 0.56, 0.75, 0.81 and 0.94 for distinguishing the basic from the R, C, S and K conditions respectively. These values indicate the degree of agreement between the class label assigned by the algorithm and the true label, after taking into account the role of chance. These values indicate moderate agreement for the R classification, substantial agreement for the S and C classification and finally almost perfect agreement for the K classification.

The above results demonstrate that the SVM algorithm is capable of exploiting the differences in the EMG activities for the different conditions. The lowest accuracy for discriminating the B and R conditions indicate that the overlap in EMG activities in greatest between these two conditions.

### Postural muscle EMGs are equal to or better that focal muscle EMGs for discriminating all constrained conditions

**Figure 5:**
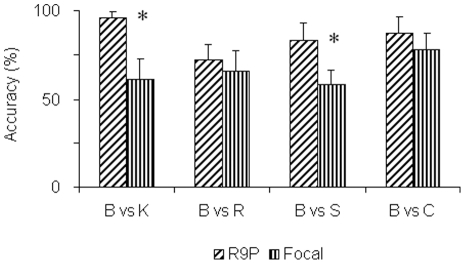
Postural vs focal muscle classification. A comparison of the capacities of the R9P subset of postural muscle vs focal muscle EMG data for the binary classification of the unconstrained B condition against the constrained K, R, S and C movements. The figure shows that the discrimination obtained using postural muscle data was as much or higher than what was obtained using the focal muscles. Discrimination capacities of the postural muscle EMGs were significantly higher for discriminating the K and S conditions (* *p*<0.01, ANOVA, *Tukey HSD* posthoc).

In order to compare the inter-condition modifications of the postural muscles with those of the focal muscles, we undertook the classifications tasks described above using each isolated data subset i.e. by the creation of a *focal* or *postural muscle vector*. There were a greater number of postural than focal muscles in the tests that produced figure 4 viz. 9 focal muscles and 15 postural muscles. This discrepancy had to be removed as we compared the capacity for discrimination in each muscle subgroup. In order to do this, the postural muscle vector in each classification was created by randomly picking the data from 9 postural muscles (denoted by R9P). If we just used information from the postural muscles, our ability to distinguish the totally non constrained movements from the constrained movements was 84±0.13% (mean ± std) for all conditions. In the case of the focal muscles, it was 66±0.14%. Taking the individual cases (Figure 5), the capacity of the postural muscle vectors for discriminating the unconstrained and constrained movements were found to be significantly higher in the K and S conditions (*p*<0.001, ANOVA, Tukey HSD). The higher mean accuracies using the postural muscle vectors in the C and R conditions were not found to be significant.

The higher overall capacity for classification using the postural muscles cannot be attributed to differences in the EMG amplitudes of the two types of muscles as these values were normalized for each individual and muscle (The EMG amplitudes were not normalized for each condition individually).

These results show that between conditions, there are discriminable differences between the postural muscle EMGs. The results also show that in some conditions, these discriminable differences are greater than those that are present for the focal muscles. When classifying the B and S conditions for example, the focal EMGs for the two conditions are sufficiently alike to wrongly classify the focal muscle EMGs in more than 30% of the test cases. In fact, more accurate classifications as to whether a B or S movement was made can be obtained by analyzing the postural muscle EMGs. It is not altogether unexpected that a greater discrimination would be obtained using postural muscle EMGs when the constraints were applied at the postural level. It is however surprising that this would be the case especially in the S conditions where the constraints were at the focal level.

**Figure 6:**
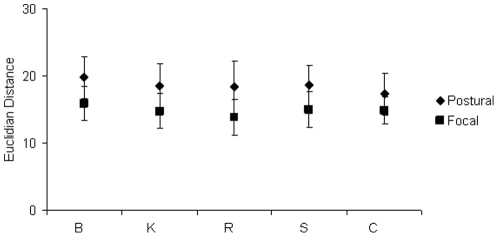
Intra class variability of focal and postural muscle EMGs. A comparison of the Euclidean distances of postural and focal muscle vectors from their mean. The Euclidean distances for the postural muscles were found to be significantly higher than that of the focal muscles in every condition (*p*<0.01, ANOVA, *Tukey HSD posthoc*). The higher Euclidean distance of the postural muscles demonstrates that their intra class variability was higher than that of the focal muscles.

A final test was carried out in order to probe if a higher variability had contributed to the poor classification capacities of the focal muscles. This was done with the use of Euclidean distances. For this test we used the *focal* and *postural muscle vectors* that had been used for the results displayed in figure 5. For each condition, we computed the mean of the *focal* and *postural muscle vectors*. The Euclidean distances of each *focal* or *postural muscle vector* from their respective means were then calculated. This permitted us to obtain an idea concerning the variability of the vectors representing each group. The mean Euclidean distance for the postural muscles was found to be significantly higher than that for the focal muscles for the B, K, R, C and S conditions i.e. in every experimental condition (Figure 6). This result showed that for every condition there was a higher intra group variability for the *postural muscle vectors* than for the *focal muscle vectors*.

### A comparison of the discriminative capacities of some of the postural muscle EMG variables

In the section above, we had demonstrated that higher accuracies for identifying the movement that had been performed could be obtained by using *postural muscle vectors* than the *focal muscle vectors*. In this section, we will describe the results of tests conducted to compare the importance of certain postural muscle EMGs variables for this discrimination. In order to determine the relative importance of certain temporal and amplitude factors of an EMG burst in the encoding of these movements, we attempted to classify the different movements by using these extracted features from the postural muscles. For these tests, the input vectors to the SVM consisted of either vectors made up of the maximum amplitude of the EMG for each muscle (*maximum amplitude vector*) or vectors that contained information concerning the time at which the EMG burst maximum occurred (*maximum time point vector*).

Table 1 illustrates that the categorization success with these reduced vectors was much less than with the full EMG vectors of the previous two sections. It is very important to note that the results in Table 1 were reported as accuracies above chance levels. This is in contrast to the manner in which they are presented in the other graphs where accuracy is presented as the total percentage of correct answers. This was done in order to highlight the different classification capacities of these two vector types. In most cases, the categorization success using the maximum time point vector was close to chance. The exception to this was the K condition discrimination in which the *maximum time point vector* was able to discriminate between constrained and unconstrained conditions with an accuracy that was approximately 17% above chance.

For all classifications, the mean accuracy with the *maximum amplitude vector* was higher than what was obtained using the *maximum time point vector*. The accuracy rates obtained using the *maximum amplitude vectors* were significantly higher than those using the *maximum time point vector* when discriminating the unconstrained movements from the S and C conditions *(p<0.01*, *Friedman test*, *Wilcoxon posthoc*, *Bonferroni correction)*.

These results show that when executing such whole body pointing movements, the time at which the postural muscle EMGs attain their maxima, is quite similar between movements. The exception to this is the K condition. Since for all conditions, a higher mean classification was obtained using the *maximum amplitude vector*, our results also show that the maximum EMG amplitude rather than the time at which it occurs should be the first variable considered when describing how the body adapts to various constraints during whole body pointing.

### Classification with individual postural muscle EMGs in the B vs S discrimination

Since the role of the postural muscles was the most surprising in the classification of the B vs S condition, we undertook further analyses in order to understand which muscles had contributed the most to this classification. This would help us to understand the manner in which postural muscles adapted themselves for such a constraint. An advantage to using a machine learning technique in this step was the ability to use the entire EMG waveform of each muscle for the classification. Table 2 displays the mean classification accuracy that was obtained using individual muscles. The accuracies were arranged in decreasing order from top to bottom.

**Table 2:** Individual postural muscle classification accuracies.

Postural muscle	Mean classification accuracies (%)
Per	73
VL	69.32
VM	67.03
SM	66.69
RF	66.07
Tib	65.88
BF	57.00
GM	55.00
ES	55.90
OI	53.38
Sol	52.72
Gast	52.78
AL	52.11
RA	45.35
ST	44.70

Mean classification accuracies obtained when using individual postural muscle EMGs for a B vs S classification. Muscles are named using abbreviated forms. Full names may be obtained in the Methods section.

Once again the results demonstrate that the classification accuracy from using information from any one muscle was less than what had been obtained using all 15 postural muscles. This affirms once again that the B vs S classification displayed in figure 5 had been obtained in a multivariate manner. The results from Table 2 also demonstrate that all discrimination accuracies above 60% had been obtained from muscles of the legs rather than those of the trunk. These muscles were the peroneus (Per), vastus lateralis (VL), vastus medialis (VM), semimembranosus (SM), rectus femoris (RF) and Tibialis (Tib). Accuracies obtained from all the trunk muscles were close to chance levels. These muscles were the erector spinae (ES), internal oblique (IO) and rectus abdominus (RA). These results indicate lower discriminable differences between the EMGs of the proximal trunk muscles between the B and S conditions.

An accuracy of over 60% was not obtained from the EMGs of all leg muscles. The following muscles also gave mean classification accuracies below 60%: biceps femoris (BF), gastrocnemius (gast), adductor longus (AL), soleus (Sol), gluteus maximus (GM) and the semitendinosus (ST).

## Discussion

In the following sections we will discuss the results that had been obtained above. We were able first of all to show that the SVM is capable of classifying constrained from unconstrained whole body pointing pointing movements with their high number of degrees of freedom. This was a prerequisite for its exploitation in identifying ensemble differences between constrained and natural movements. In the case of every type of constraint, the postural muscle EMGs were as or more discriminative of the movement condition than the focal muscles. This suggests that the modest alterations in the visible movements of the postural module which had been evaluated by earlier studies using kinematic methods [13] were in fact the resultant of very active muscular changes at the postural level. Such discriminative alterations support the hypothesis that the role of the postural muscles is not just one of maintaining equilibrium but also one of actively fulfilling the task demands by transporting the focal module towards the target. When further probing the high discriminative capacities of the postural muscles between the B and S conditions on a muscle by muscle basis, we observed a poorer performance from the trunk muscles than from several leg muscles. This suggests that a higher overlap in EMG activity is to be found in the trunk muscles during adaptations of the postural muscles to the S condition. While vectors containing information on the timing of EMG maxima were unable to discriminate the movement conditions, vectors containing extracted EMG amplitude information consistently gave accuracies above chance, hence suggesting that amplitude rather than timing was adjusted to tune the whole body pointing movement for constraints.

Further discussions of these results will be presented in the sections that follow. In particular we make some general comments on the use of machine learning techniques. This includes mention of some pitfalls that must be avoided when this approach is employed. Finally we propose some steps that might be taken to make the use of such techniques more common practice.

### SVMs are able to discriminate EMG data from different kinds of whole body pointing

In this study we carried out a multivariate ensemble analysis of the muscular activity underlying the complex multijoint activity of whole body pointing. As movement requires the involvement of several muscles, each of which can be described by several variables, studies on motor control could benefit from these techniques. A univariate comparison of the variables in this study using techniques like the *ANOVA* [30], [31] would have involved the comparison of at least 72 different variables (24 muscles×3 EMG variables). One way of reducing the size of this task is to create meaningful ensembles of muscular activity and then to carry out a comparison of the ensembles. Sometimes, the analysis of group activity may also reveal features that may not be apparent at the individual level. An example of this in the current study was what we had observed in the tests using collective data from postural muscles as opposed to that from focal muscles.

The first goal of our investigation was to determine if the SVM was able to discriminate between the unconstrained and constrained movements. One of our previous studies had shown that kernel methods are able to distinguish the gait of arthritic and control subjects [8]. As opposed to gait however, the whole body pointing task involves an active participation of the focal module and its interactions with the postural module. Gait is thought to involve primarily the rhythmic activity of the neurons in the spinal column while the whole body pointing task is a goal oriented task including the focal module and the involvement of the cerebral cortex [5], [6]. It was uncertain if the SVM would be able to distinguish these whole body pointing movements involving the active participation of many more joints and a higher number of degrees of freedom. Figure 4 illustrates that the SVM was capable of distinguishing the unconstrained movements from the constrained movements with a mean success rate close to 80% or above irrespective of whether these constraints were focal or postural. The capability of the SVM to retrieve information concerning the altered EMGs can also be seen in the values of the κ coefficients associated with these classifications. They indicated moderate to almost perfect agreement between the labels assigned by the SVM and the actual class to which the movement belonged. These results therefore illustrated the potential of these machine learning techniques in uncovering discriminable differences between the variants of the whole body pointing movement. This capacity is poorest for the R condition, indicating that the EMGs from these movements showed the highest overlap with the unconstrained movements.

The non negative matrix factorization method that had been used earlier had uncovered what was in common for the activities of these 24 muscles during the different movement conditions viz. a tri-dimensional organization [14]. Any modification were relatively difficult to quantify using this type of dimensionality reduction techniques because (1) data are projected on the subspace resulting in difficulties for retrieving information and (2) intra/inter group variability can induce significant noise about the extracted muscle synergies. Our results demonstrate that despite a relatively unchanged covariation [14], [15] in the muscular activities that the imposed constraint on the basic whole body reaching (B condition) induced significant modifications, some of which are detailed in the following sections.

### Discrimination between conditions by postural muscles

Using postural muscle EMGs we were able to with the exception of the R condition, to discriminate all constrained from unconstrained movements with a mean accuracy higher than 80%. This indicates that postural muscles undergo significant adjustments in order to achieve the constrained conditions. The higher discrimination capacities of the postural muscles were especially surprising in cases where the most visible changes from the kinematic studies had been observed at the focal level. When comparing for example the B and S movements, the differences between the centre of mass trajectories were quite modest compared to changes in the hand trajectory (figure 1). This then provides one more example of a case where kinematic modifications can be quite modest while it is not so with the underlying muscle activation patterns. This has been observed with some types of gait. The kinematics were found to be basically invariant in the case of backward walking or walking with various loads while the accompanying muscle activation patterns were found to be quite altered in each case [32].

It has to be emphasized that the higher discriminative capacities of the EMGs from the postural muscles may not be attributed to a higher amplitude of these EMGs. This variable was normalized so that the maximum EMG amplitude of each muscle was one. Information concerning the differences in amplitudes between conditions however was available as the normalization was done over all conditions.

As a high variability is one factor that could contribute to difficulties in classification, we used the Euclidean distance from the mean as a measure of intra class variability in the case of the *postural* and *focal muscle vectors*. As these vectors were the normalized vectors that had been used for classification, the higher Euclidean distances for the *postural muscle vectors* indicated a higher intra class variability for these vectors than for the focal ones.

The higher inter class discriminative capacities of the postural muscles along with their higher intra class variability suggest that these muscles undergo greater modifications than the focal muscles for achieving the different movements. It may be expected that the postural muscles are better able to differentiate the experimental conditions like K with a postural constraint. It is however surprising for the S conditions, in which constraints existed at the focal level. We might ask why a similar result was not obtained for the C condition. This is primarily due to the higher discriminative capacities of the *focal muscle vectors* in the C condition. The mean classification accuracy of these vectors in the C condition was 80.1% as opposed to 58% in the case of discriminating the S condition. In fact, the mean classification accuracy using the *postural muscle vector* was higher in for the C condition than for the S. It was 88.6% for the C condition as opposed to 83.6% for the S (figure 5).

The fact that postural adjustments accompany movements in the focal module is known [33]. Indeed these adjustments commence even before movement is detected in the focal module. These postural EMG changes seen at the very earliest stages of the movement have primarily been thought to play a role in maintaining equilibrium [34]. Our study sheds new light on these modifications by illustrating the discriminative nature of the physiological activities underlying these postural adjustments even when they appear modest following kinematic analyses. Indeed a higher level of postural EMG activity may be required to maintain the lower variance observed at the kinematic level.

This level of changes at the postural level may indicate a double role that has to be fulfilled by the postural muscles. Indeed many researchers have suggested that postural adjustments during a whole body reaching task are not just a compensation for mechanical disturbances but play an active role in moving the arm towards the target [35]–[37]. In other words the postural module may also have a focal role. Our results indicating that the postural EMGs undergo alterations that correspond to different focal constraints provide some support for this hypothesis.

From a neurophysiological point of view, several lines of evidence suggest that there is a hierarchical organization of motor command in which kinematic goals (here, hand trajectories) are specified mainly at higher levels in the hierarchy of the CNS and are translated into kinetic motor commands mostly at lower hierarchical levels. Thus goal directed movements would be planned in terms of a kinematics framework (for review see [38]). Our finding that postural muscle activity reflects arm trajectory suggest that the motor command connecting the motoneurons in the spinal circuitry may be encoded in a global/kinematic context. Kinematic goals specified at higher levels in the chain of motor command may not only be translated into a pattern of muscular activations at the focal level but also at the postural level [32], [39]. Postural muscle activity would therefore represent the output of the motor command controlling the body geometry to generate a precise hand displacement toward the target.

The idea that this double role played by the postural muscles is partly responsible for the important modifications in the postural muscles receives some support from the results in Table 2. The figure shows that the highest discrimination between conditions was obtained from muscles of the lower limb rather than those of the trunk. The former rather than the latter muscles are closer to the ground and in a position to play the double role of exerting the mechanical forces that would be required for maintaining equilibrium as well as moving the centre of mass and finger closer to the target.

### Classification between B and S conditions using individual postural muscle EMGs

Table 2 displays the classification accuracies from attempts to classify the individual postural muscle EMGs as belonging to a B or S movement. They reveal that all the postural muscles giving mean classification accuracies over 60% are muscles of the leg. As opposed to this all the trunk muscles were able to discriminate with lower accuracies. As in the previous cases, this indicates a good deal of overlap in the EMGs of the trunk muscles between constrained and unconstrained conditions.

Among the leg muscles, accuracies over 60% were obtained from the peroneus, tibialis, vastus lateralis, vastus medialis, semimembranosus and rectus femoris. The first two are flexors of the ankle. The ankle extensors, the soleus and gastrocnemius on the other hand, gave poorer performances suggesting that around the ankle, antigravity activities might be more general and less tuned to each type of movement. It has previously been suggested in association with the Hufschmift phenomenon that antigravity activities might be more general [40], [41]. Around the knees however, the muscles that performed better were made of extensors (VL, VM) as well as biarticular muscles (RF, SM).

Further studies are required to find if the same pattern of tuning holds for the other pointing movements.

### Maximum amplitude or timing

Once we had affirmed that postural muscles constitute the more discriminative module in these movements, we went on to identify which characteristics in these EMGs were better able to discriminate between conditions. For comparison, we picked the maximum amplitude of each EMG (*maximum amplitude vector*) and the instances at which these had occurred (*maximum time point vector*). The latter parameter was picked using theoretical considerations as well as what was known considering the order of these muscle bursts from a previous study [14]. Since the classification is based on the Euclidean distance of any input vector from the surface separating the two classes, any displacement of the moment at which the input vector maximum occurred could play a critical role in the classification. We found in each case that the mean discrimination obtained using the *maximum amplitude vector* was higher than with the *maximum time point* vector (Table 1). In order to highlight the differences in the results obtained with the two variables, we reported the discriminative capacities above chance levels (50%).

A first observation from these results is that the accuracies using these extracted features are lower than what was obtained with the full EMGs. This demonstrates clearly that a multivariate approach is useful in discriminating the EMGs coming from the different movement conditions. More variables important in distinguishing the two groups of EMGs, was present in the full EMG vectors.

Our results obtained using the *maximum time point* vector are in accordance with what had been observed by Chiovetto et al [14]. They had reported using the same EMG activities that are currently under investigation, that all 24 muscles had a triphasic organisation in time. The order of muscle bursts was the same for all the variants of whole body pointing. The study of Ivanenko et al [17] had also emphasised the role of timing as a central organizing principle for the activating patterns of the leg muscles as subjects combined locomotion with other voluntary movements.

Since discrimination capacities using amplitude are above chance for all movement conditions, our results suggest that this is one of the variables that is tuned in order to achieve the different constrained movements. This is in agreement with the muscle synergy hypothesis, according to which neural networks in the spinal cord may be able to recruit muscle synergies and scale them in amplitude to generate a large repertoire of motor tasks from a small number of motor primitives [17], [42].

### Multivariate techniques and general conclusion

Multivariate techniques have been increasingly in use in the field of Neuroscience [43]–[45] in order to understand population encoding. They can be extremely useful in the field of motor control where it is necessary to comprehend the activities of a large number of variables. Indeed the use of the SVM in this study permitted us to observe collective features that may have escaped classic univariate analyses.

Another advantage of the use of the SVM can be seen in the classifications that were done using individual muscles. The discrimination between conditions was done using the entire EMG of each muscle. Classic EMG comparisons are usually carried out using single features such as maximum amplitudes, onset timings, offset timings etc. If there are insufficient differences between any of these variables, a multivariate analysis would be able to incorporate the information from several variables. A classification using the entire EMG waveform also avoids the difficulties involved in defining and extracting any specific feature such as onset time. The utility of a multivariate classification in discriminating these conditions can be clearly seen by comparing the classification accuracies in figure 4 as opposed to those in Tables 1 and 2. Lower accuracies were obtained in the latter two cases where less features were incorporated in the vectors used for classification.

The insights obtained in developing such a methodology can also be applied to any complex physiological data. This is not only true for multiunit neuronal recordings but also for surface recordings such as multi channel evoked potentials. As the emphasis of such a technique is on the identification of differences between datasets, it is not limited to such time series but can be extended to any 2 dimensional datasets. In this category we would not only include images from fMRI but also results from molecular biology such as in situ hybridisation where it is necessary to compare patterns. Advantages to be obtained from using machine learning techniques such as the ability to combine features for arriving at a conclusion or avoiding the definition of a particular feature would apply to these fields as well. Indeed it is already possible to find the application of such techniques in the field of in situ hybridisation [46] and fMRI analysis [47].

Some important precautions however have to be taken into account when using SVMs or any other machine learning techniques. Since during the training phase optimization techniques are being used to create a nonlinear separating surface between defined datasets, it is possible to find separating surfaces that only hold for the used examples. In this manner, it may even be possible to classify noise. This surface however, would be unable to correctly separate exemplars that had not been used for the training. For this reason it is important to set aside for the testing phase, data that had not been used for training. In this manner the problem of false classifications will be avoided. The technique of systematically separating the data set into training and testing sets is called cross validation.

Further studies that could be carried out in this domain would be testing the use of simpler methods such as linear discriminant analysis (LDA) [48] with the data presented in this study. We had begun with the SVM because of their proven efficiency in previous studies [8]–[10]. The widespread use of machine learning methods however might require the availability of methods that are easier to implement. The LDA method is considerably simpler to understand than the SVM. Even though lower classification accuracies may be obtained by using LDA, the accuracies achieved may be sufficient for achieving a comparative study between various muscle groups.
